# A liberal strategy of red blood cell transfusion reduces cardiovascular complications in older patients undergoing cardiac surgery

**DOI:** 10.1186/cc13297

**Published:** 2014-03-17

**Authors:** R Nakamura, JL Vincent, J Fukushima, J Almeida, F Bergamin, C Park, E Osawa, M Sundin, A Muller, F Galas, L Hajjar

**Affiliations:** 1 Instituto do Cancer do Estado de São Paulo, Brazil; 2 Erasme Hospital, Université libre de Bruxelles, Brussels, Belgium

## Introduction

Owing to their high risk in cardiac surgery, it is essential to define which transfusion strategy results in a lower rate of cardiovascular complications in older patients [1]. The aim of this study was to compare clinical outcomes after the implementation of either a restrictive or a liberal transfusion strategy in patients aged 60 years and above.

## Methods

This study was a substudy of the Transfusion Requirements After Cardiac Surgery study. In this subgroup analysis we included all patients aged 60 years and above randomized to a restrictive or a liberal strategy of RBC transfusion. A composite endpoint for cardiovascular complications was used and defined as a combination of 30-day allcause mortality and severe cardiovascular morbidity.

## Results

The primary composite endpoint - all-cause 30-day mortality, cardiogenic shock, or myocardial infarction - occurred in 9.6% of patients in the liberal strategy group and in 18.4% in the restrictive strategy group (*P *= 0.041). The incidence of cardiogenic shock was 5.2% in the liberal group and 12.8% in the restrictive group (*P *= 0.031) and of myocardial infarction was 2.2% in the liberal group and 5.6% in the restrictive group (*P *= 0.203). There was no significant difference between transfusion strategies in 30-day mortality rates (4.4% vs. 8%, respectively; *P *= 0.23).

## Conclusion

In this prospective, randomized clinical trial, older patients submitted to a restrictive strategy of RBC transfusion had a rate of cardiovascular complications in 30 days after cardiac surgery twice as high than a liberal strategy. In this group of patients, probably untreated anemia would be more harmful than in a younger or healthier population undergoing cardiac surgery.
